# The Management of Elbow Instability Using an Internal Joint Stabilizer: Preliminary Results

**DOI:** 10.1007/s11999-014-3646-2

**Published:** 2014-05-01

**Authors:** Jorge L. Orbay, Michael R. Mijares

**Affiliations:** The Miami Hand and Upper Extremity Institute, 8905 SW 87th Avenue, Suite 101, Miami, FL 33176 USA

## Abstract

**Background:**

Nonsurgical and surgical treatments such as immobilization, transarticular pinning, and hinged or nonhinged external fixation have been used to treat unstable elbows. These methods all have drawbacks. We thought that a bent Steinmann pin introduced through the axis of ulnohumeral rotation and attached to the ulna could provide an improved method of treatment and that this could result in the development of a proper internal joint fixator that may have widespread application.

**Questions/purposes:**

Does a fully internal hinged fixator crafted intraoperatively by the surgeon from a Steinmann pin for patients undergoing surgery for severe elbow instability result in restoration of range of motion and elbow stability? Does it result in new complications?

**Methods:**

We reviewed the first 10 patients treated with the method for elbow instability. Diagnoses included fracture-dislocations of the elbow that remain unstable after fracture repair and unstable elbows that result from release of contracture or ulnohumeral synostosis. During that time, all patients meeting these criteria who underwent surgery by this surgeon (JLO) were treated with this approach. Charts, radiographs, and therapy notes were assessed at a minimum of 14 months (mean, 32 months; range, 14–59 months); no patients were lost to followup. Data recorded included age, sex, and elbow and forearm range of motion as well as any complications and reoperations that occurred. The absence of elbow instability was determined initially by radiographically observing concentric reduction of the ulnohumeral and radiocapitellar joints and later by radiography plus the absence of clinical signs and symptoms of elbow instability.

**Results:**

Mean range of motion at latest followup was flexion 134°, extension −19°, pronation 75°, and supination 64°. All elbows were clinically and radiographically stable. Complications resulting in additional procedures occurred in four patients, including one recurrent deep infection in a patient with a remote history of sepsis, one wound hematoma that resolved after a drainage procedure performed in the office, one prominent implant treated by partial removal, and one patient with heterotopic ossification treated with excision of the heterotopic bone.

**Conclusions:**

This technique restores elbow stability and permits motion without the use of transcutaneous pins. It seems promising for the treatment of patients with severe elbow instability but requires a second procedure for removal. Further investigation is needed to understand its place in the surgeon’s toolbox and what drawbacks it may have.

**Level of Evidence:**

Level IV, therapeutic study. See Guidelines for Authors for a complete description of levels of evidence.

## Introduction

Managing the unstable elbow after injury or surgical release is often difficult [13, 16]. Elbows with complex fracture-dislocations such as terrible triad injuries and unstable coronoid fractures can remain unstable after fracture repair [7, 8]. Elbow dislocations with severe soft tissue injury and those in morbidly obese patients may be unstable after reduction. Elbows subject to reconstructive procedures where it is necessary to perform extensive release of periarticular soft tissues and/or excision of heterotopic bone often become unstable, hindering or delaying rehabilitation. In the past, several methods of temporary stabilization have been used to manage elbow instability. Immobilization in flexion has been the first line of defense and is accomplished by splinting or casting for a period of time sufficient for soft tissue healing. This method is not always effective because flexion may fail to provide sufficient stability, especially in obese patients, and prolonged immobilization may lead to elbow stiffness [9, 12]. Transarticular pinning was often used in the past but presents the additional drawback of damaging the articular surface. Hinged external fixators have been advocated but these seldom provided the desired early motion as a result of difficulty in aligning to the axis of ulnohumeral rotation and a high prevalence of pin tract pain and infection [1, 3, 4, 7, 11–15]. Nonhinged external fixators are currently favored for their simplicity but they totally prevent joint motion, are clumsy, and still present pin tract problems [4, 6, 15].

**Fig. 1A–B:**
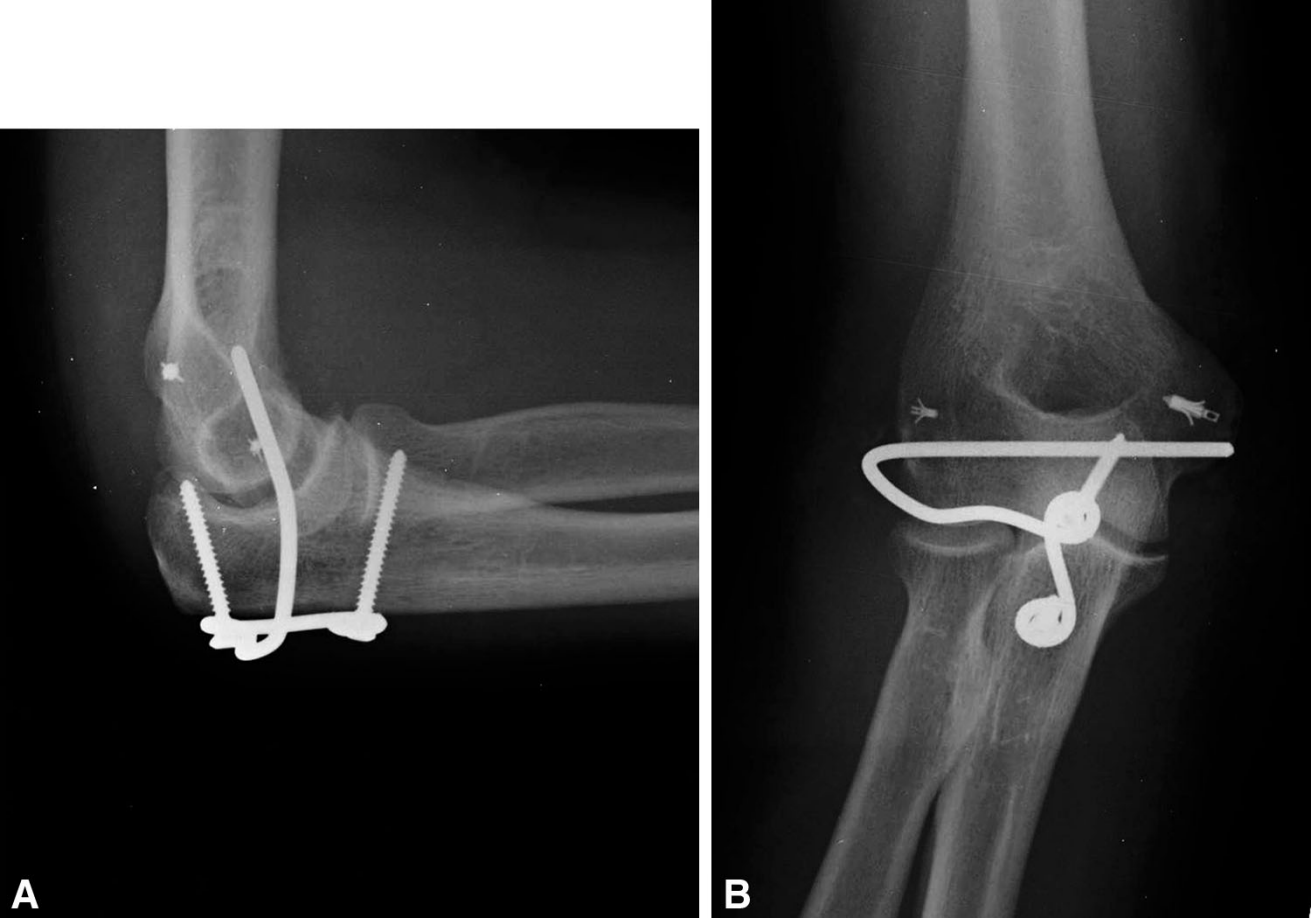
AP and lateral radiographs show the joint stabilizer. (A) The implant is attached to the ulna by means of two 3.5-mm compression screws. (B) The axis portion of the implant traverses the distal humerus in line with the axis of ulnohumeral rotation.

**Fig. 2:**
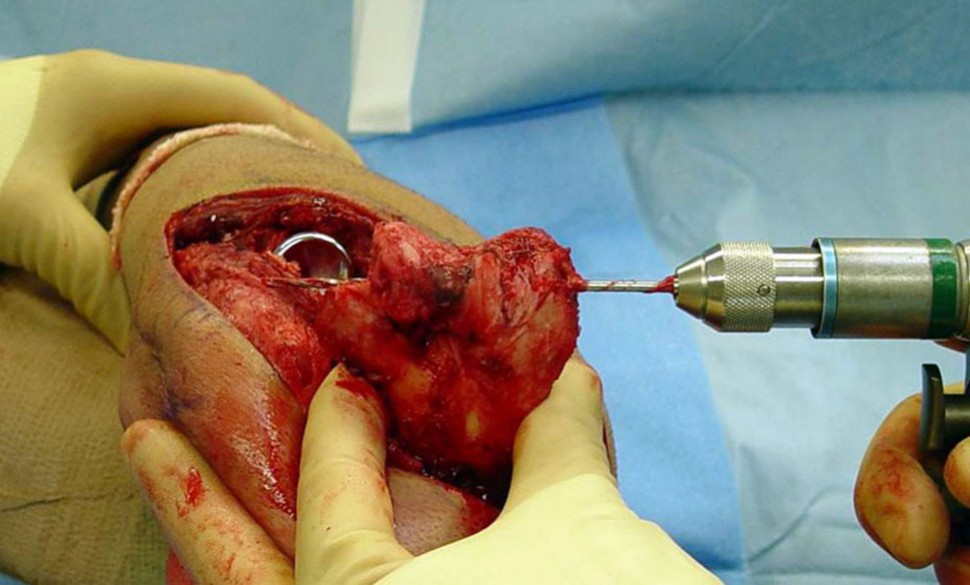
In this case, the axis of ulnohumeral rotation could be determined by directly visualizing the origin of both collateral ligaments.

**Fig. 3:**
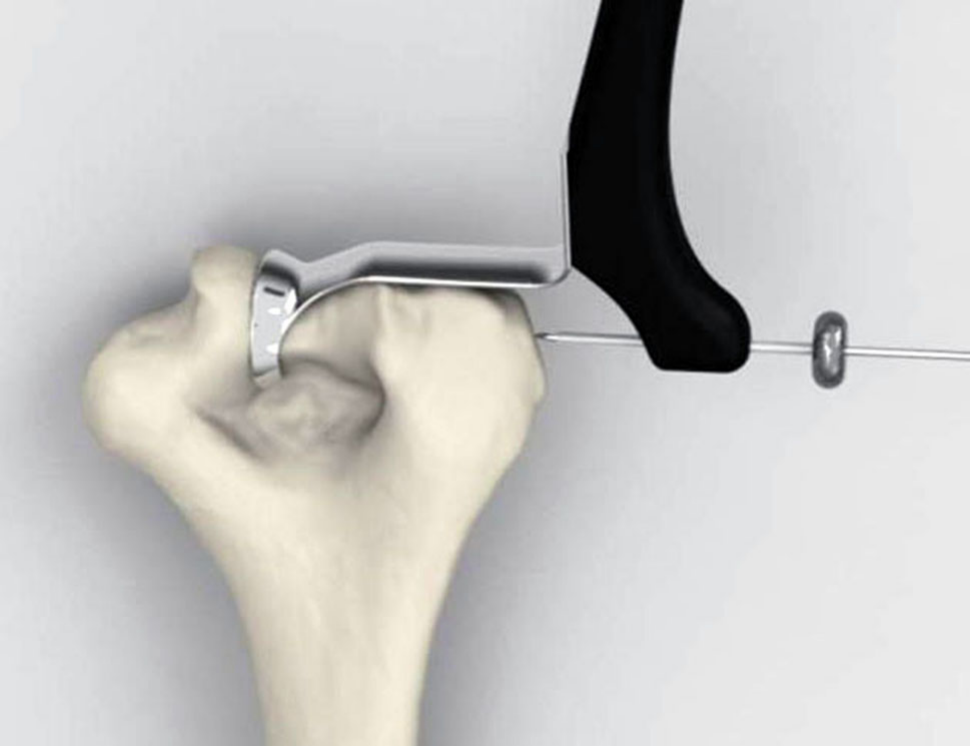
We developed a guide to facilitate location of the axis of ulnohumeral rotation through a single lateral approach. The guide defines a medial point on this axis by centering on the medial trochlear expansion. The surgeon determines the lateral point on the axis by locating the anatomical center of the capitellum. A Kirschner wire inserted along these two points establishes the axis.

We have proposed and used a low-profile totally implanted joint stabilizer for the management of elbow instability (Fig. 1A–B). This fully implanted device is intended to prevent redislocation and allow early motion while avoiding the difficulties inherent to other methods. In this series, we created the elbow joint stabilizer implant by carefully shaping a 2.5-mm Steinmann pin. A straight section of the implant (the axis pin) is inserted into a hole drilled along the axis of ulnohumeral rotation. A line between the origins of both collateral ligaments reveals the axis (Fig. 2). Also, after several cases had been performed, we developed a special guide to assist the surgeon in accurately determining the location of this axis when exposure is limited to one side of the humerus (Fig. 3). Axis pin rotation inside the humerus permits elbow flexion/extension; motion in any other plane is prevented by rigidly attaching the other end of the implant to the ulna using compression screws. This device is implanted temporarily to allow for ligamentous healing and is intended to be subsequently removed in a simple secondary surgical procedure.

In the present report, we sought to determine whether this fully internal and simple hinged fixator, crafted intraoperatively, for patients undergoing surgery for severe elbow instability results in restoration of ROM and elbow stability. Does it result in new complications?

## Materials and Methods

We performed a review of the first 10 patients we treated with the elbow stabilizer implant from June 2008 to November 2009. Inclusion criteria were acute traumatic elbow instability not manageable with immobilization in flexion, persistent instability of a terrible triad injury after surgical repair, the need to neutralize tenuous fixation of an unstable coronoid fracture, and elbow instability resulting from the surgical release of a fused elbow. During that time, all patients meeting these criteria who underwent surgery by this surgeon (JLO) were treated with this approach; no other treatments were used to treat those kinds of elbow instability during this time. Minimum followup was 14 months (mean, 32 months; range, 14–59 months). No patients were lost to followup.

**Fig. 4A–C:**
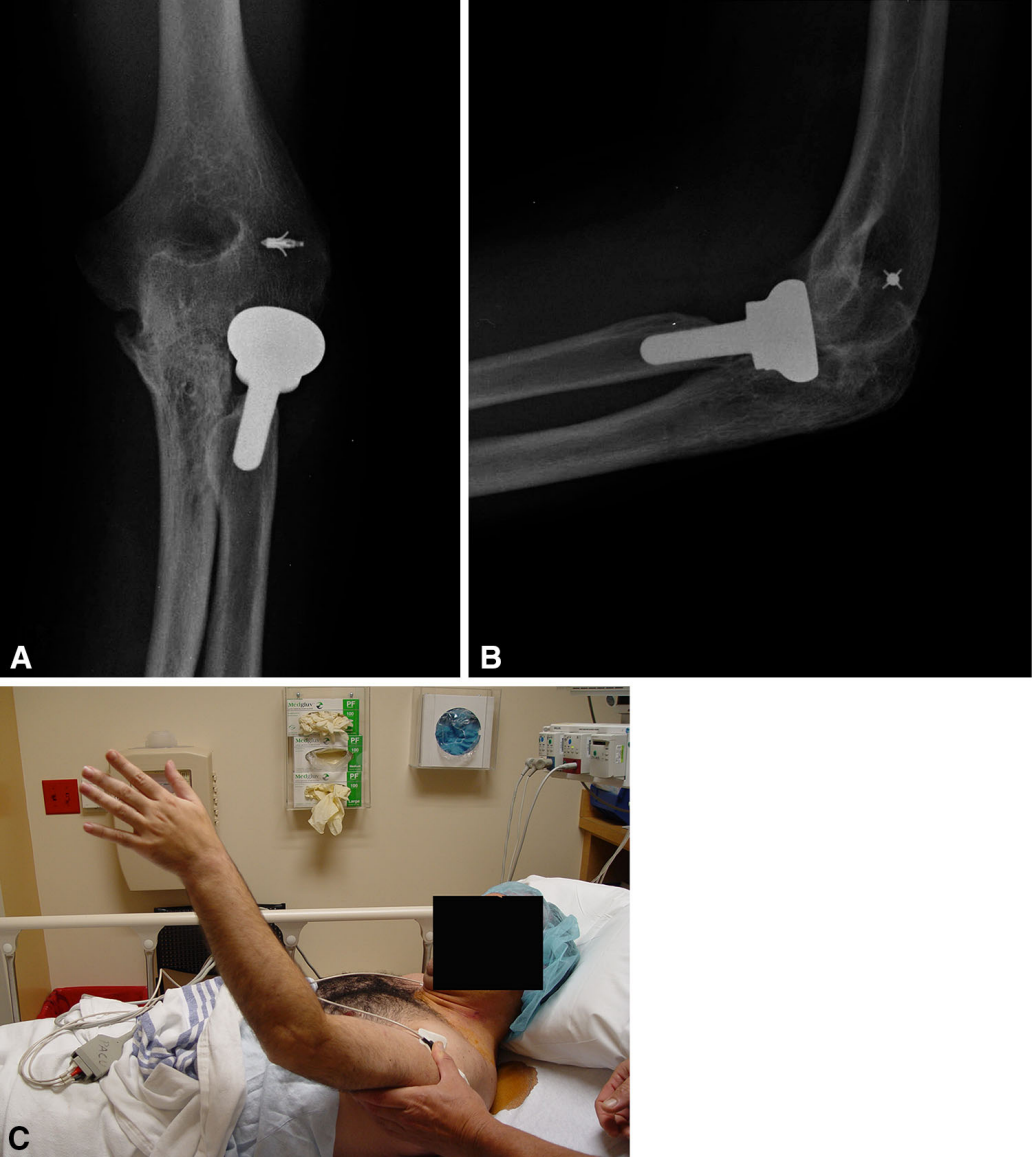
A 38-year-old man presents with ulnohumeral synostosis after complex trauma and multiple surgeries. (**A**) A preoperative AP and (**B**) lateral view are shown. (**C**) The elbow is fused in 45° of extension.

**Fig. 5:**
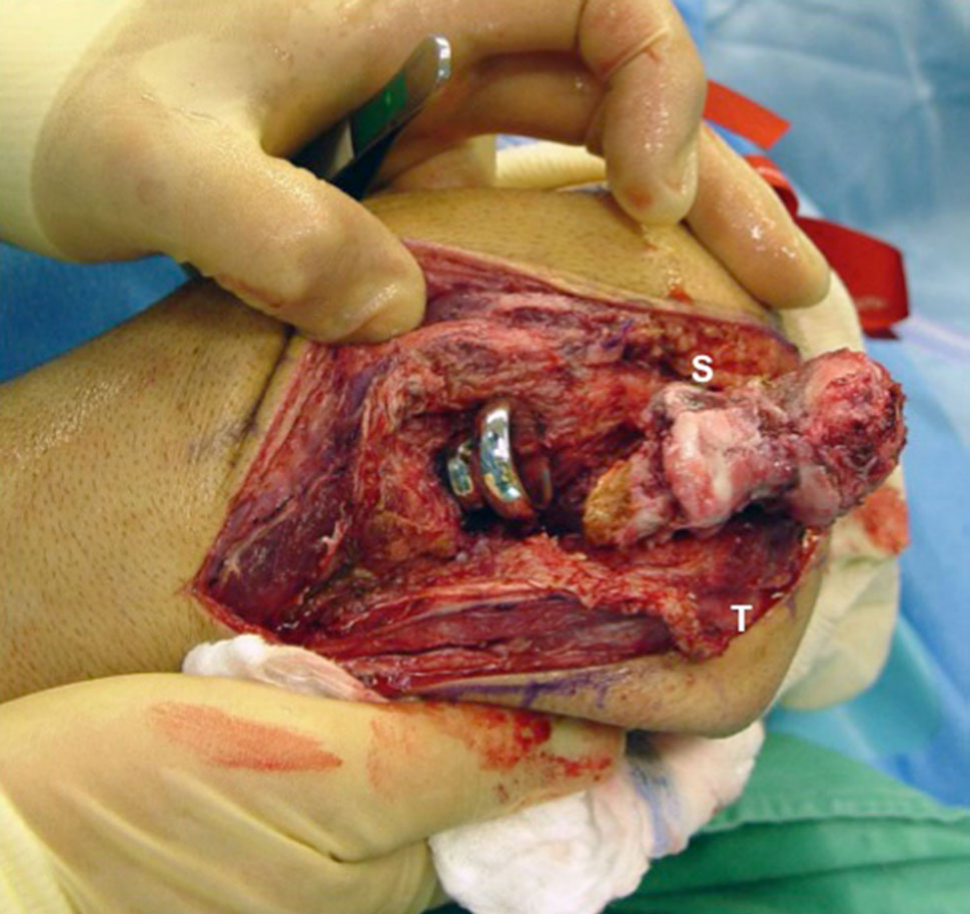
Extreme instability resulted after completion of surgical release and takedown of an ulnohumeral joint synostosis (S) on the anterior medial aspect of the trochlea. Reworking of both articular surfaces was necessary. Both collateral ligaments had to be released, but the triceps (T) and brachialis were kept intact. In this case, the collateral ligaments were ossified and were not present after bone resection.

Five patients had acute injuries: three were acute elbow dislocations with radial head and coronoid fractures that proved unstable when placed in flexion after repair or had redislocated after repair. One was an unstable posterolateral elbow dislocation in an elderly obese female that had dislocated multiple times after closed reduction. One presented with a medial elbow dislocation and an unstable fracture of the medial facet of the coronoid. Five patients had chronic problems: four had posttraumatic stiffness, heterotopic ossification, and/or ulnohumeral synostosis with a mean 10° arc of flexion/extension (Fig. 4A–C), and one had developed ulnohumeral synostosis in full extension associated with juvenile rheumatoid arthritis. These last five cases all resulted in unstable elbows after surgical release (Fig. 5). There were six males and four females with a mean age of 43 years (range, 9–67 years) at the time of surgery. Seven unstable elbows involved the left extremity and three involved the right.

**Fig. 6A–D:**
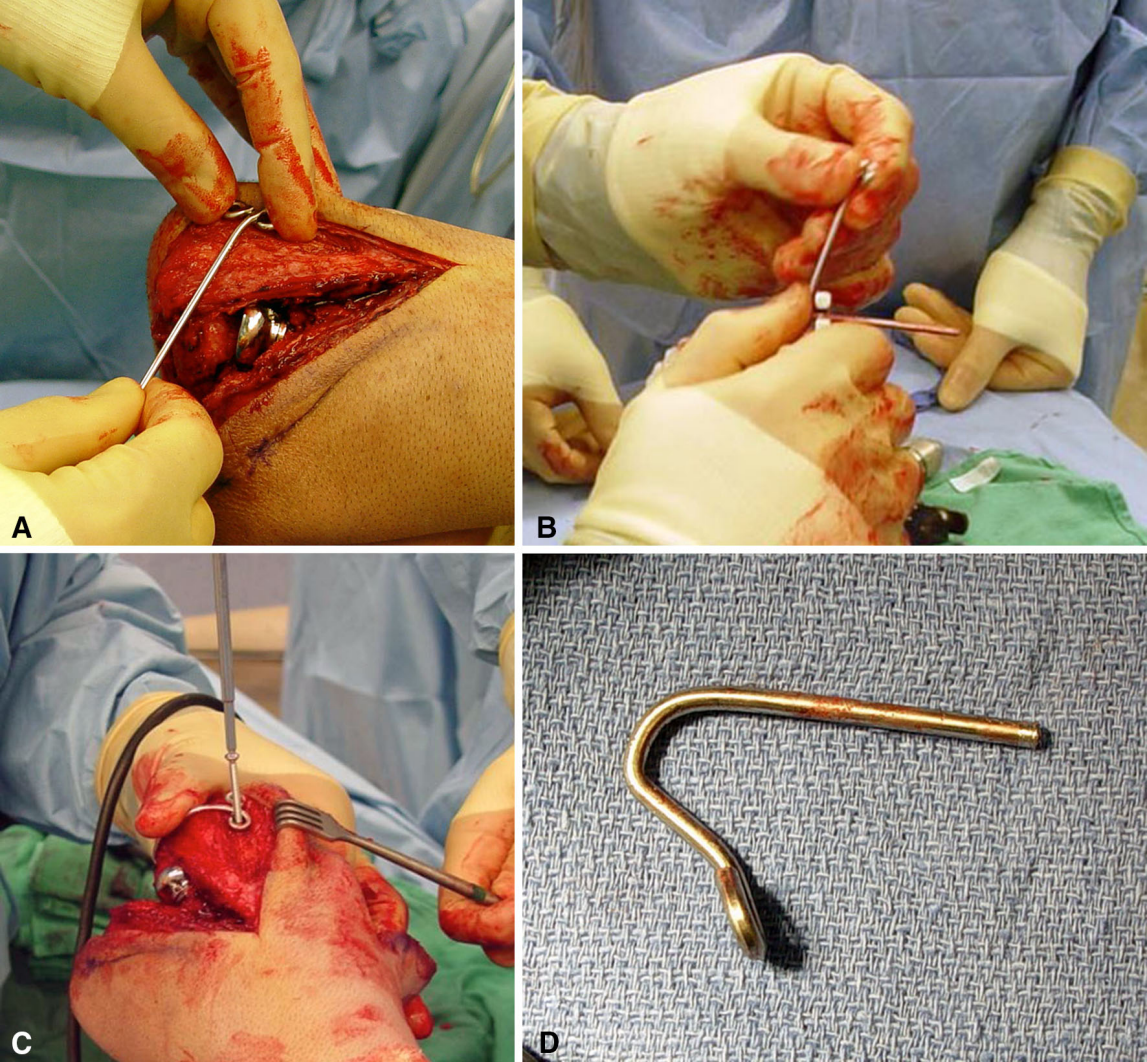
The joint stabilizer is created from a 2.5-mm Steinmann pin. (**A**) A figure-of-eight section is first formed on the blunt end to accept two screws for attachment to the ulna. (**B**) The axis portion is established by making a sharp bend at the proper location and then cut to the appropriate length. (**C**) The pin is now contoured to the ulnar surface and secured, after concentric elbow reduction, by means of two 3.5-mm screws. (**D**) The final shape of the internal joint stabilizer is shown.

**Fig. 7A–B:**
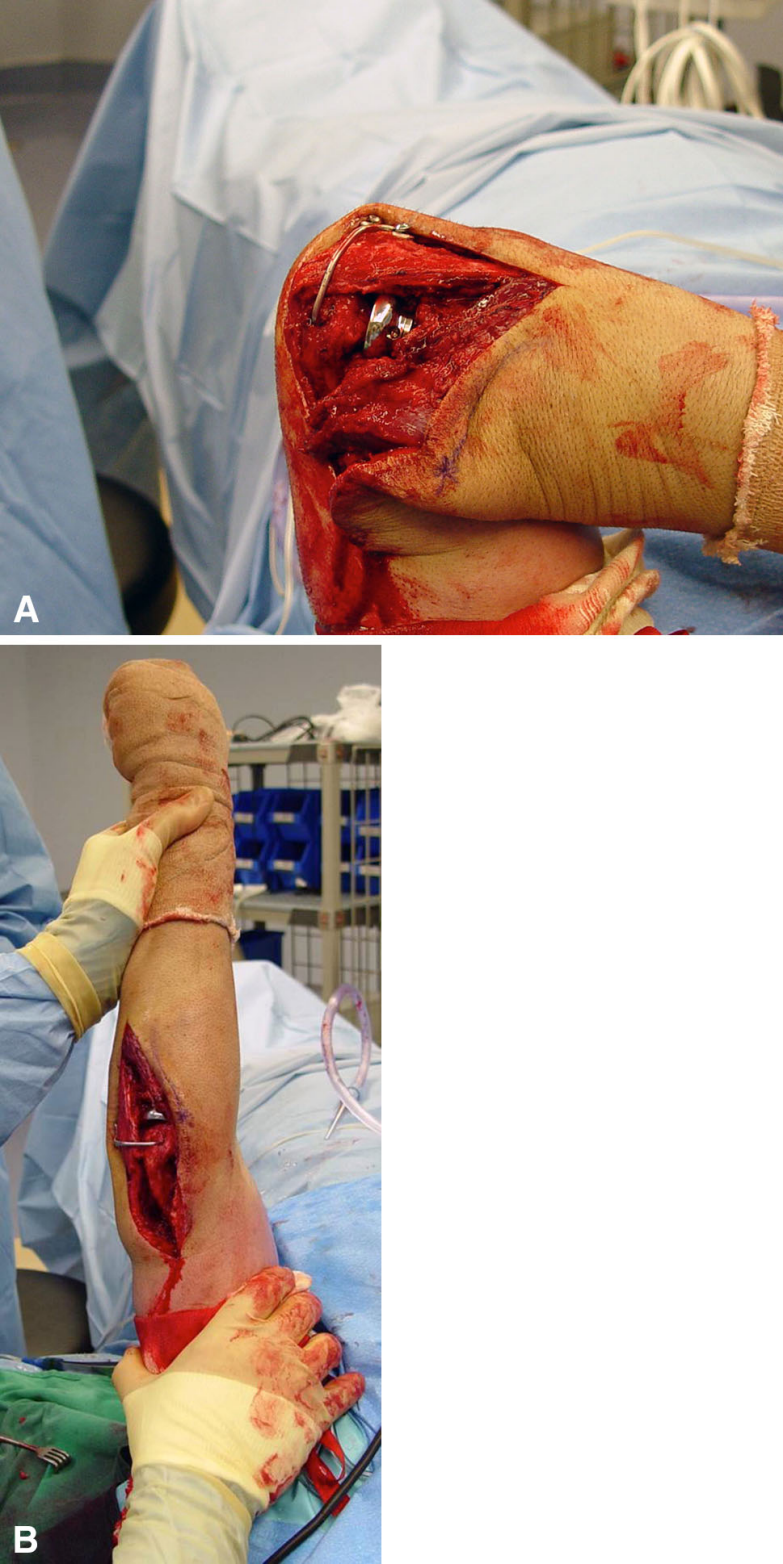
(**A**) Intraoperative assessment was performed for restoration of flexion, (**B**) extension and stability in all directions.

All surgical procedures were performed by the same surgeon (JLO) either in an ambulatory or hospital setting and subsequently managed at the Miami Hand and Upper Extremity Institute. When the need for the joint stabilizer was determined preoperatively, the implant was premanufactured out of a 2.5-mm Steinmann pin by bending the ulna attachment portion into a figure-of-eight shape. This preworked Steinmann pin was then sterilized and its remaining straight portion cut and shaped intraoperatively to fit the patient’s anatomy. When the need for using a joint stabilizer was perceived intraoperatively, the device was then created fully intraoperatively by bending a Steinmann pin into the figure-of-eight shape around a 3.5-mm compression screw using two strong pliers and cut and bent to fit the patient’s anatomy (Fig. 6A–D). Full intraoperative manufacturing added to operating room time (estimated 1 hour, including placement). Restoration of elbow flexion/extension and stability in all directions were assessed intraoperatively before wound closure (Fig. 7A–B).

**Fig. 8:**
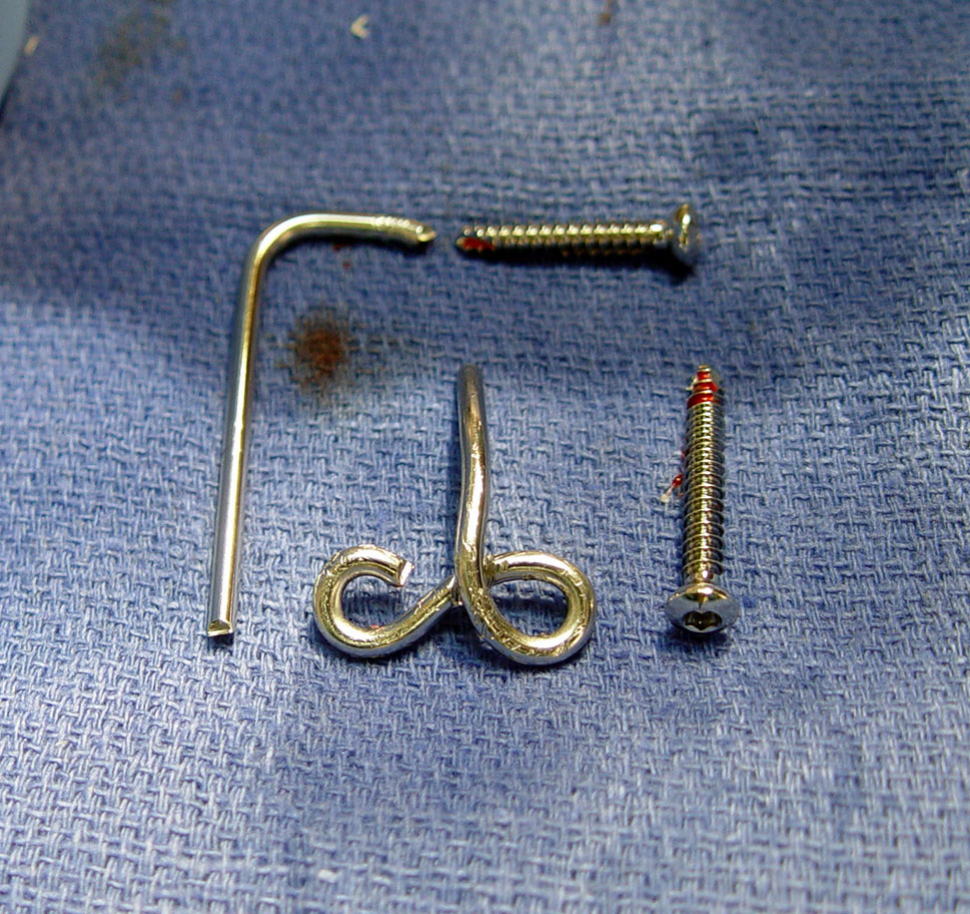
The device is removed after 6 to 8 weeks.

Patients were seen 1 week after the index procedure and on a monthly basis until therapy was discontinued. They were then seen 6 months later and asked to return yearly. Unprotected elbow motion was allowed after the first postoperative visit. We planned to surgically remove the device after the first 6 weeks when soft tissue healing was expected to maintain stability. The joint stabilizer was in place for a mean time of 7 weeks in eight patients. Two patients refused to have their implants removed, because they did not wish to return to the operating room and reported having no pain or discomfort relating to the implant. Both of these patients have had their implant in place for 5 years and have been evaluated at 58 and 59 months. They were found not to have problems or radiographic changes. This is not the intended course and implant breakage could subsequently occur. Removal was done by making two small incisions, allowing removal of the axis pin end of the Steinmann pin with a wire cutter and the ulnar end of the pin by removing the two screws (Fig. 8).

**Fig. 9A–B:**
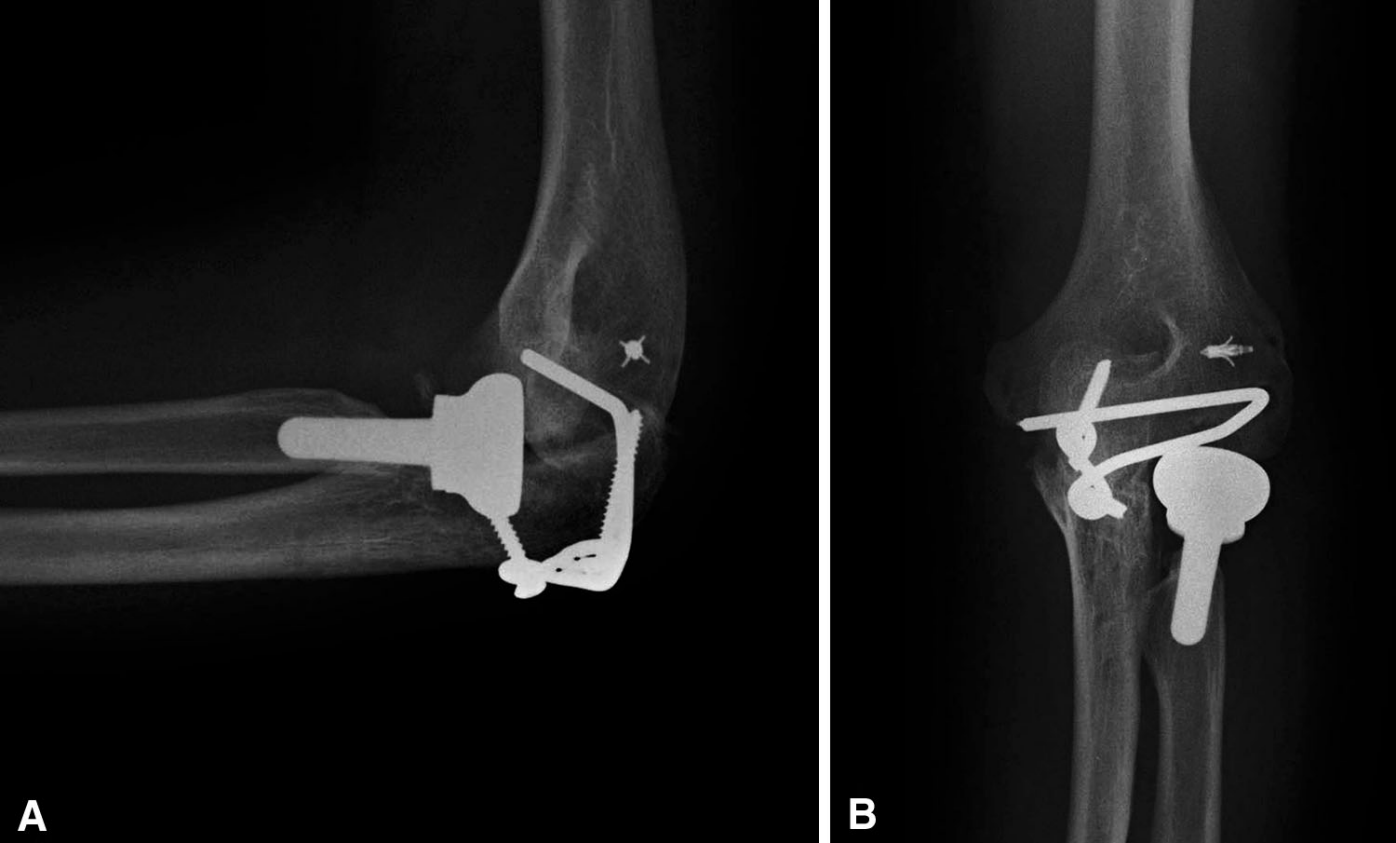
(**A**) Lateral and (**B**) AP radiographic views of the joint stabilizer are shown.

During followup visits, we evaluated elbow motion including flexion-extension, pronosupination, finger motion, and radiographic alignment (Fig. 9A–B). All patients received a long-arm postoperative dressing in 90° of flexion. This dressing was usually kept on for 1 week. At 1 week, the wounds were inspected and all patients were then referred to therapy. At this time, patients received a removable long-arm custom-fabricated thermoplastic orthosis in 90° of flexion, forearm in neutral, and wrist and digits free. This orthosis was removed for hygiene and ROM exercises and by the third week, it was usually discarded. Therapy initially consisted of active and active assisted motion exercises, edema control, scar management, pain modalities, and home program exercises performed at least four to five times per day. Strengthening activities are initiated with isometrics followed by concentric and eccentric exercises usually at 6 to 8 weeks, once adequate ROM has been achieved. Occasionally, static progressive or dynamic splinting was used to manage delayed recovery of flexion, extension, and/or forearm rotation (in our series, four received static and two received dynamic splints).

Data were obtained by reviewing medical records, therapy records, and radiographic records. The second author (MRM) measured elbow ROM at full flexion, extension, pronation, and supination with a goniometer at final followup for each patient. Elbow stability was assessed by the operating surgeon (JLO) and elbow instability was defined as any radiographic evidence of subluxation or dislocation; the presence of catching, clicking, or popping on elbow motion; and symptoms of giving way. Complications were ascertained by chart review.

## Results

**Fig. 10A–D:**
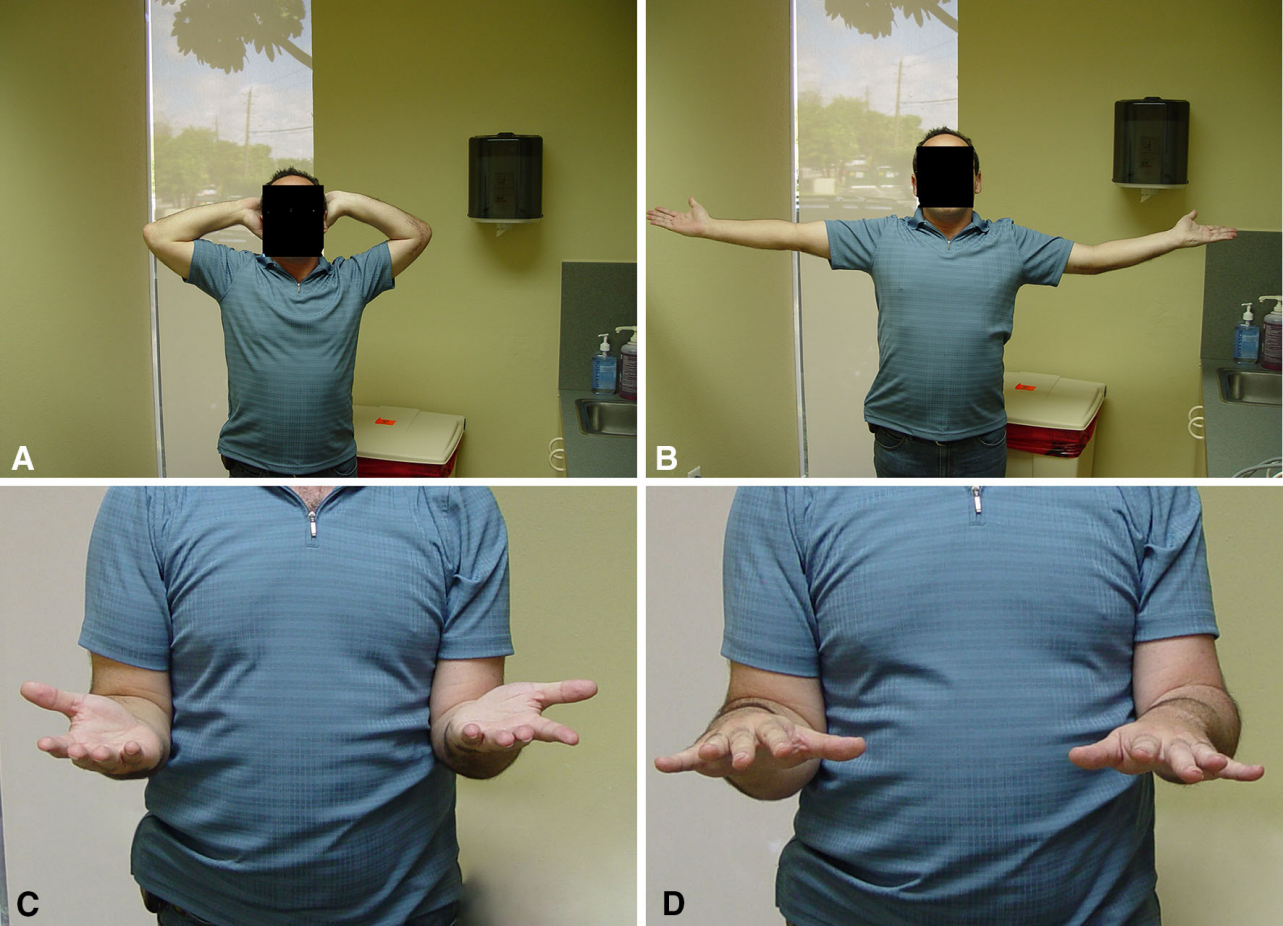
Functional ROM observed at the 1-year followup showing (**A**) flexion, (**B**) extension, (**C**) supination, and (**D**) pronation.

The average motion at a mean 32 months after surgery was flexion 134°, extension −19°, pronation 75°, and supination 64° (Table 1). Patients had a mean flexion arc of 115° and a mean pronosupinatory arc of 138° (Fig. 10A–D).

**Table 1:** Results

Descriptive statistics	Elbow flexion (degrees)	Elbow extension (degrees)	Forearm pronation (degrees)	Forearm supination (degrees)
Mean value	134	−19	75	64
Median value	135	−17	80	80

There were no patients with recurrent elbow instability. All patients presented radiographic evidence of concentric reduction of the ulnohumeral and radiocapitellar joints, no patient reported instability or giving way of the elbow, nor did any of the patient’s elbows demonstrate instability in clinical examination.

**Fig. 11A–F:**
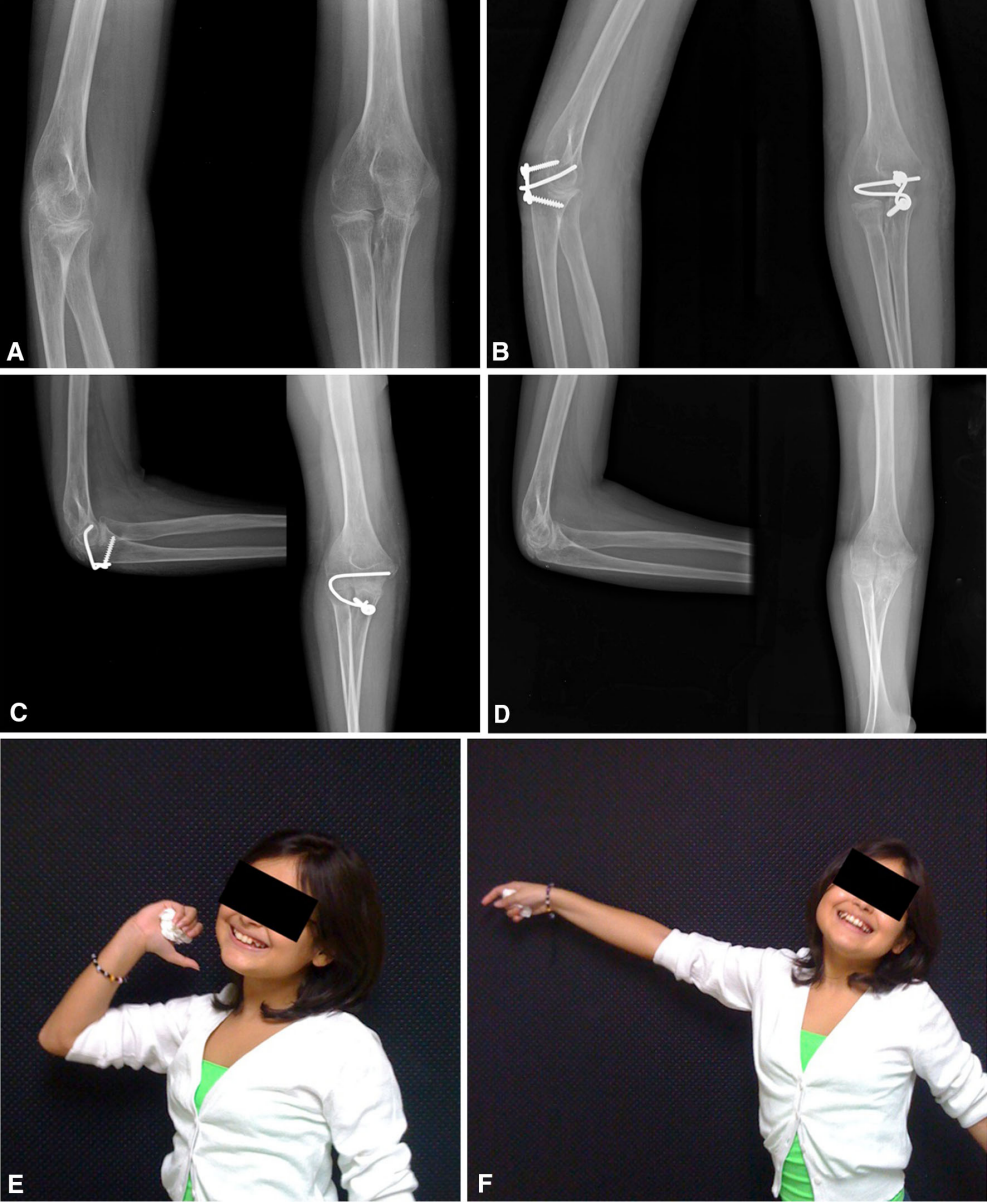
(**A**) This is a preoperative radiograph of a 9-year-old girl who has juvenile rheumatoid arthritis with 6 months’ duration of complete loss of elbow motion. The elbow was fixed in full extension, having failed all forms of conservative treatment. (**B**) After full release and takedown of an early synostosis, the elbow was stabilized with the joint stabilizer implant. (**C**) At 4 weeks, a painful proximal screw head and implant prominence were delaying rehabilitation; therefore, the proximal screw and proximal part of the implant were removed. (**D**) Radiographic images of the elbow 2 years after the final removal of the implant are shown. (**E**) Elbow flexion and (**F**) extension are shown 2 years postimplant removal.

Complications were identified in four patients, all four of whom had additional procedures to manage the complications. One patient had a superficial wound hematoma that resolved with suture removal and simple drainage in the office. A second patient was a small 9-year-old child with juvenile rheumatoid arthritis who developed pain over the prominent proximal part of the ulnar implant attachment on elbow flexion, therefore limiting rehabilitation. This resulted in the premature removal of the offending part of the implant (Fig. 11A–F). A third patient developed Stage 3 heterotopic bone formation after a terrible triad injury, which was treated with surgical excision and release. The fourth patient, with a history of osteomyelitis, developed a recurrent deep infection of the elbow. This problem resolved after hardware removal, surgical drainage, and treatment with intravenous antibiotics.

## Discussion

The management of the unstable elbow, whether resulting from acute trauma, its sequelae, or chronic disease, has often proven difficult [13, 16]. All methods of elbow stabilization to date are associated with serious drawbacks; any method that prevents ulnohumeral motion delays rehabilitation and may result in stiffness. Steinmann pins through the joint damage articular cartilage, external fixation can result in pin tract problems, and hinged external devices can be difficult to apply and uncomfortable for patients. As a result of the lack of suitable alternatives, we introduced this method. In this article, we sought to determine whether this fully internal and simple hinged fixator crafted intraoperatively by the surgeon for patients undergoing surgery for severe elbow instability would result in restoration of ROM and elbow stability. Also, we investigated whether its use resulted in new complications.

This study has several limitations. Our sample size is small, this was a retrospective study, and the procedures were performed by only one surgeon; these results will need to be confirmed in larger series by individuals not involved with the development of this technique to verify its generalizability. Our small sample size reflects the fact that severely unstable elbows are not common and most patients with elbow instability can be managed successfully with conventional methods. Further research will need to compare the new approach with conventional treatments. Followup was short, but success in managing elbow instability generally resides in preventing early redislocation. Redislocation after the first year is uncommon.

The joint stabilizer provided sufficient clinical stability to start early rehabilitation. All patients started unsupported motion exercises and were allowed active use of the extremity for light activities of daily living immediately after their first postoperative visit. Both McKee et al. [7] and Yu et al. [15] report that hinged external fixation also allowed for immediate motion; Sørensen and Søjbjerg [12] make no mention of this. Our final mean arcs of ulnohumeral motion and forearm rotation are similar to those of the other reported series. The mean final arc of flexion-extension in our series was 115°, McKee et al. [7] reported 105°, Sørensen and Søjbjerg [12] 95°, and Yu et al. [15] 93°. Our pronation-supination arc was 138°, whereas McKee et al. [7] reported 105°, Yu et al. [15] 96°, and Sørensen and Søjbjerg [12] 108°. In view of the small numbers, these are similar, but comparative studies are needed to know this with certainty. In all our patients, the use of the implant resulted in a concentrically reduced elbow with no evidence of clinical instability. We identified 11 previous studies on the use of hinged external fixators for elbow instability (Table 2). McKee et al. [7] reported in their series that one of their 16 patients had recurrent instability; Sørensen and Søjbjerg [12] had one patient out of 17 with persistent subluxation, and Yu et al. [15] reported one of 20 with loss of articular reduction.

**Table 2:** Literature review

Study (year)	Total number of subjects	Mean followup (months)	Flexion-extension arc	Flexion	Flexion contracture	Pronation-supination arc	Pronation	Supination
Cobb and Morrey [2] (1995)	7	Minimum 24 (24–84)	88° (57°–110°)	124° (87°–140°)	36° (20°–65°)	116° (70°–140°)	61° (30°–75°)	55° (10°–70°)
McKee et al. [7] (1998)	16	23 (14–40)	105° (65°–150°)	127°	22°	151°	76° (20°–90°)	75° (15°–90°)
von Knoch et al. [14] (2001)	11	9 (2–33)	81° (50°–125°)	104° (71°–130°)	23° (5°–40°)	–	–	–
Ruch and Triepel [11] (2001) Group 1: acute Group 2: > 6 weeks after original injury	3	6 (4–10)	120° (105°–130°)	–	25° (20°–30°)	–	90° (All 90°)	67° (44°–90°)
	5	6 (4–10)	84° (75°–95°)	–	33° (15°–50°)	–	68° (45°–90°)	43° (0°–70°)
Jupiter and Ring [4] (2002)	5	38 (12–98)	123°	136° (120°–140°)	13° (5°–20°)	Full	Full	Full
Pugh et al. [10] (2004)	36	34 (20–65)	112° ± 11°	131° ± 11°	19° ± 9°	136° ± 16°	–	–
Yu et al. [15] (2007)	20	25 (12–85)	93° (30°–145°)	114° (70°–145°)	22° (0°–60°)	96° (0°–180°)	52° (0°–90°)	44° (0°–90°)
Lindenhovius et al. [5] (2008)	18	29 (10–53)	119° (75°–145°)	135° (120°–145°)	17° (0°–45°)	141° (70°–180°)	78° (45°–90°)	63° (20°–90°)
Zeiders and Patel [16] (2008)	32	36 (12–60)	100° (30°–130°)	130°	12° (0°–20°)	–	–	–
Egol et al. [3] (2007)	29	27 (12–105)	109° ± 57°	–	–	128° ± 44°	–	–
Sørensen and Søjbjerg [12] (2011)	17	44 (12–83)	95° (40°–125°)	129° (110°–150°)	34° (15°–75°)	–	–	–
Our results	10	32 (14–59)	115° (60°–140°)	134° (90°–150°)	19° (5°–35°)	138° (70°–180°)	75° (34°–90°)	64° (10°–90°)

Ranges shown in parentheses.

Complications are frequent with all methods used to treat the unstable elbow (Table 3). McKee et al. [7] reported six complications out of 16 patients, three of whom required reoperation. These included instability, pin tract infections, chronic regional pain syndrome, superficial wound infection, and transient radial palsy. Sørensen and Søjbjerg [12] reported seven of 17 patients, did not mention reoperation rate, and found subluxation, pin tract infection, deep infection, pin hole-related humeral fracture, transient ulnar palsy, or permanent ulnar palsy. Yu et al. [15] reported 10 complications out of 20 patients, did not mention reoperation rate, and found subluxation, heterotopic ossification, pin tract infection, adhesive shoulder capsulitis, or aseptic pin loosening. We had four complications in the 10 patients, all resulting in additional procedures. Comparative studies are called for to determine which approach will be most successful, generalizable, and safe.

**Table 3:** Complications

Study (year)	Complications	Reoperation (%)
Cobb and Morrey [2] (1995)	Fracture at proximal ulna through distal pin hole secondary to fall (14%) Pin migration (14%) Persistent instability (14%)	14
McKee et al. [7] (1998)	Pin tract infections in the humerus (13%) Posterior subluxation of elbow in the external fixator (6%) Reflex sympathetic dystrophy (6%) Superficial wound infection (6%) Transient palsy of the radial nerve (6%)	19
von Knoch et al. [14] (2001)	Pin tract infection (45%)	0
Ruch and Triepel [11] (2001)	Arthrofibrosis (13%) Minor ulnar nerve deficit (13%) Pin tract infection (13%)	13
Jupiter and Ring [4] (2002)	Medial skin flap blistering (20%) Retained broken 5-mm external fixation pin in the humerus (20%) Transient ulnar nerve irritation associated with medial external fixation pin (20%)	20
Pugh et al. [10] (2004)	Hardware removal (11%) Radioulnar synostosis (6%) Posterolateral rotatory instability (3%) Wound Infection (3%)	22
Yu et al. [15] (2007)	Heterotopic bone formation (25%) Pin tract infection (10%) Adhesive capsulitis of the ipsilateral shoulder (5%) Aseptic pin loosening (5%) Loss of articular reduction (5%)	20
Lindenhovius et al. [5] (2008)	Ulnar nerve dysfunction (33%) Stiffness (6%) Heterotopic ossification (6%)	33
Zeiders et al. [16] (2008)	Heterotopic ossification (9%)	0
Egol et al. [3] (2007)	Minor pin drainage at proximal pin cluster (46% of the 13 of 29 patients who underwent hinged external fixation) Heterotopic ossification (34%) Resubluxation 2 weeks after external fixator removal (3%) Radial head loosened (3%)	17
Sørensen and Søjbjerg [12] (2011)	Pin site infections (18%) Ulnar nerve palsy–temporary (6%) Humeral fracture through a pin site hole (6%) Deep elbow infection leading to upper arm amputation (6%) Radial nerve palsy (6%) Permanent incomplete ulnar nerve palsy/frozen shoulder/complex regional pain syndrome (6%)	6

In conclusion, this is an alternative form of elbow stabilization that maintains a concentric reduction while permitting joint motion and therefore promoting rehabilitation. Further investigation is needed on the effectiveness and complications of the joint stabilizer as compared with other techniques such as hinged and nonhinged external fixation. Clinicians may consider this device when facing unstable fracture dislocations in patients in whom early motion is not contraindicated. The benefits of early motion must compensate for the need for a secondary removal procedure and the risk of soft tissue irritation.
